# Lower Choline Rate in the Left Prefrontal Cortex Is Associated With Higher Amount of Alcohol Use in Alcohol Use Disorder

**DOI:** 10.3389/fpsyt.2018.00563

**Published:** 2018-11-07

**Authors:** Rodrigo Stênio Moll de Souza, Marcos Rosa, Thaísa Malbar Rodrigues, Thayssa Dalla Costa Escobar, Emerson Leandro Gasparetto, Ester Miyuki Nakamura-Palacios

**Affiliations:** ^1^Department of Internal Medicine, Health Sciences Center, Federal University of Espírito Santo, Vitória, Brazil; ^2^Health Sciences Center, University Hospital Cassiano Antônio de Moraes, Federal University of Espírito Santo, Vitória, Brazil; ^3^Department of Radiology, Federal University of Rio de Janeiro, Rio de Janeiro, Brazil; ^4^BRAEN – Brazilian Research Group on Brain and Cognitive Engineering, Federal University of Espírito Santo, Vitória, Brazil; ^5^Laboratory of Cognitive Sciences and Neuropsychopharmacology, Graduation Program in Physiological Sciences, Federal University of Espírito Santo, Vitória, Brazil

**Keywords:** ^1^H MRS, choline, prefrontal, alcohol use disorder, alcohol intake

## Abstract

Excessive and long-term alcohol consumption produce metabolic changes, such as of choline, in many brain regions in alcohol use disorder (AUD) and in non-AUD subjects as well. This study examined the association of choline proportion in the prefrontal cortex with pattern of alcohol use in AUD patients. The choline metabolite was acquired through a single voxel Proton Magnetic Resonance Spectroscopy (^1^H MRS). Between-groups comparison corrected by age showed that the ratio of Choline/Creatine (Cho/Cr) was significantly smaller (*p* = 0.005) in the Left Prefrontal (LPF) of AUD patients when compared to paired non-AUD subjects. A multiple regression analysis corrected by age showed that decreasing ratios of Cho/Cr in the LPF was associated with increasing amount of alcohol consumption in drinks per day (*p* < 0.01) in AUD patients. Rates of Cho/Cr in the LPF was inversely related to amounts of alcohol consumption possibly indicating the severity of the AUD. Thus, low proportion of Cho/Cr in the LPF could indicate more severe AUD (higher alcohol intake).

## Introduction

Alcohol use disorder (AUD) is a complex disease with the association of biological, social and psychopathological components (1). Excessive and long-term alcohol consumption produce neuronal synaptic connectivity dysregulation and apoptosis (2), evidenced by volumetric reduction and functional and metabolic changes in the brain (3, 4).

Proton Magnetic Resonance Spectroscopy (^1^H MRS) is a non-invasive method capable of identifying metabolic changes *in vivo*, which has been used to analyze and compare the tissue energy metabolism of healthy and diseased individuals in different brain regions of interest (5, 6).

Cho signal measured by ^1^H MRS seems to mostly reflects the membrane turnover (7) and has been found to be altered especially in frontal areas (7, 8) but also in cerebellum (4, 9–11) and other brain regions (12, 13) in AUD patients. Changes of Cho levels in AUD has been associated with altered cerebral metabolism of lipids in membrane or myelin (8, 13), which may underlie behavioral and cognitive dysfunction seen in this condition. However, ^1^H MRS has not been stablished as a method to target alcohol use for monitoring response to treatments.

Considering that left and right prefrontal region are functionally dissociated (14, 15) the aim of this study was to examine the association of Cho/Cr of frontal regions from both hemispheres (left and right prefrontal region) with patterns of alcohol use such as the amount of daily use (drinks/day), age at onset of alcohol use, years of use and days of abstinence before ^1^H MRS acquisition in severe AUD patients. Rates of this metabolite were also compared with age and gender-matched non-AUD healthy subjects.

## Methods

### Subjects

Twenty-two male patients diagnosed with AUD were successively recruited from March 2016 to July 2017 from a specialized public outpatient service of the Medical School Hospital of the Federal University of Espírito Santo (Brazil). The control group was constituted by twenty-three healthy non-AUD age and gender-matched subjects, recruited among relatives and workers from the University Hospital from Federal University of Espírito Santo with similar socio-demographic characteristics.

This study included: (i) male patients between the age of 30 and 70 years; (ii) consuming at least 30 drinks per week on average over the previous year; (iii) with criteria for AUD according to the ICD-10 and DSM-5; (iv) in stable clinical condition with no need for inpatient care; (v) in alcohol abstinence for at least 15 days; (vi) able to read, write, and speak Portuguese; and (vii) without severe withdrawal signs or symptoms at baseline.

Conversely, exclusion criteria included: (i) a condition of intoxication or withdrawal due to a substance other than alcohol, (ii) unstable mental or medical disorder other than alcohol dependence, except nicotine and/or caffeine; (iii) a diagnosis of epilepsy, convulsions, or delirium tremens during abstinence from alcohol; (iv) a previous history of drug hypersensitivity or adverse reactions to diazepam or other benzodiazepines and haloperidol; (v) any contraindication for magnetic resonance procedures such as electronic implants, metal implants, claustrophobia, or permanent make-up or tattoo received within the previous 3 months; (vi) the presence of vascular, traumatic, inflammatory, or tumor injuries detectable by MRI examination; (vii) clinical or laboratorial diagnosis of active hepatic encephalopathy; (viii) unsatisfactory spectral curves; (ix) refuse participation in the study or to sign the informed consent form.

Ethical approval was provided by the Brazilian Institutional Review Board of the Federal University of Espírito Santo (CAAE 19403713.6.0000.5060 and 13528213.2.0000.5060), Brazil. The study was conducted in strict adherence to the Declaration of Helsinki and is in accordance with the ethical standards of the Committee on Human Experimentation of the Federal University of Espírito Santo, ES, Brazil, where this study was conducted. Subjects were fully informed about the experimental protocol and voluntarily signed an informed consent form before the start of the study.

They were clinically evaluated, interviewed regarding their socio-demographic characteristics and pattern of alcohol and drug use, including the application of the AUDIT (Alcohol Use Disorder Identification Test) (16).

### MRI acquisition

All subjects underwent MRI in 1.5 T scanner (Philips Medical Systems Nederland B.V., The Netherlands) with specific head coil model SENSE (8 channels) from University Hospital, in rest and without paramagnetic contrast administration.

Axial TSE FLAIR T2-weighted images were acquired with the following parameters: TR/TE/TI: 11000/140/2800; slice: 5 mm; acquisition matrix: 244 x 512; and sagittal 3D T1-weighted images with the following parameters: TR/TE: 8.8/4; slice: 1 mm, Matrix: 240 x 240.

### ^1^H MRS acquisition

**Figure 1:**
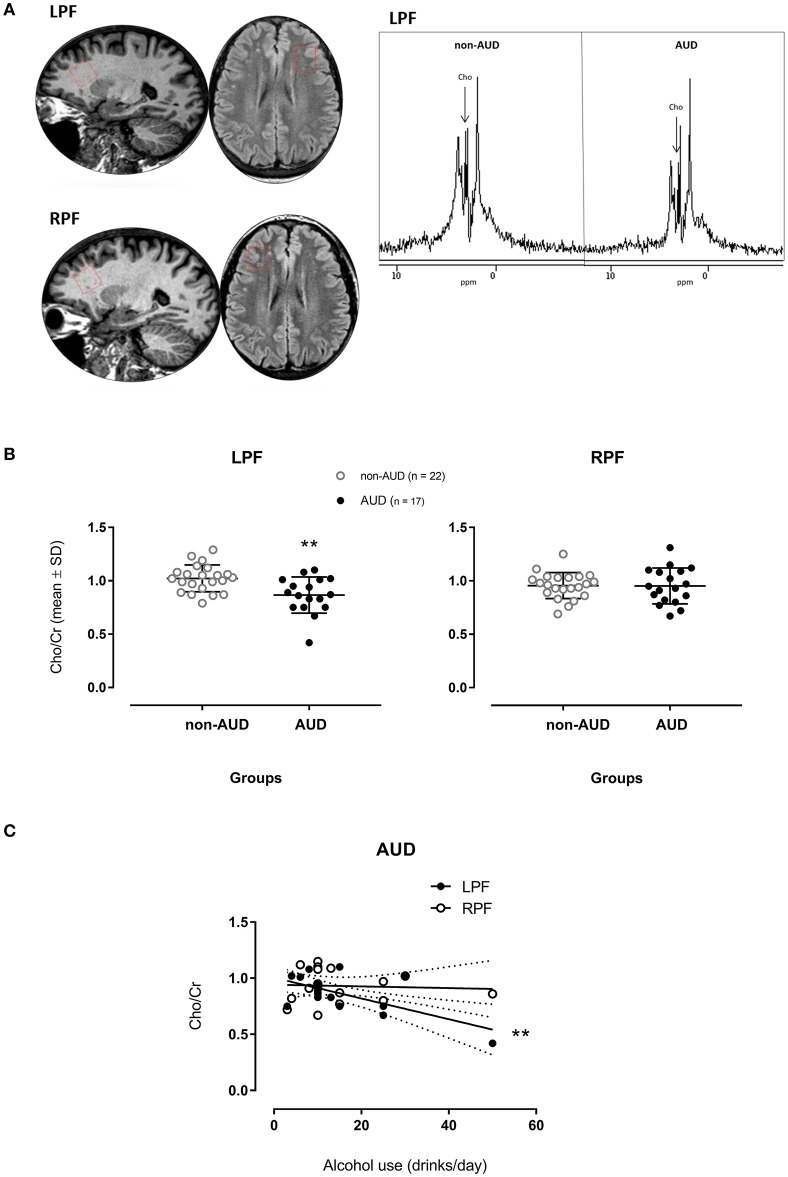
Spectrograms of one patient diagnosed with Alcohol Use Disorder (AUD) and one non-AUD subject in voxels places in the left (LPF) and right (RPF) prefrontal regions **(A)**; relative concentrations of Choline (Cho)/creatine (Cr)] measured in voxels placed in the LPF and RPF of AUD patients (*n* = 17) and non-AUD controls (*n* = 22). **p < 0.01 compared to controls (MANOVA corrected by age with Bonferroni's correction for multiple comparisons) **(B)**; Cho/Cr measured in voxels placed in the LPF in AUD patients related to the amount of alcohol use (drinks/day) **(C)** **p < 0.001 (Multiple Linear Regression Analysis corrected by age).

Single voxel spectroscopy was acquired in two voxels with epicenters located in the respective areas: left prefrontal (LPF) and right prefrontal (RPF) (Figure 1A), with the following parameters: point resolved spectroscopy sequence (PRESS); axial acquisition plane; TE: 30 ms; TR: 6 s; NEX 2; number of acquisitions: 64; Bandwidth 4 kiloHertz; spectral resolution: 4,096 points; water spectrum: not suppressed (absolute quantification); voxel size: 8 cm^3^ (2 × 2 × 2 cm).

### Post-spectral processing

After image acquisition, spectroscopy data were processed using the MRUI-AMARES software version MRUI 5.2 released on May 25, 2015, which uses a non-linear-least squares (NLLS) quantification algorithm, using relative analyzes between metabolites, with creatine as denominator.

During the processing of the spectroscopies, unsatisfactory spectral curves were identified, and their exclusion was necessary. Additionally, outliers of metabolic data defined by labeling rule with 2.2 as multiplier according to Hoaglin and Iglewicz (17) were also excluded. Therefore, five subjects from AUD and one from non-AUD group had to be excluded from analysis.

### Statistical analysis

Data are presented as the percentage or mean ± standard deviation (*SD*). Proportions of Cho over Cr from left (LPF) and right (RPF) prefrontal cortex were compared between groups (non-alcoholics vs. alcoholics) by a multivariate analysis of variance (MANOVA) corrected by age (data were normally distributed according to D'Agostino & Pearson normality test), and a *p*-value lower than 0.025 was considered statistically significant according to Bonferroni's multiple comparisons correction considering two comparisons (one metabolite ratio in two regions of interest). Effect size was calculated using Cohen's d and corrected by Hedges's *g*_*s*_ for unpaired *t*-tests (18).

Cho/Cr from AUD patients in LPF and RPF were introduced as independent variables in multiple regression analyses corrected by age having amount of alcohol use in drinks/day, age at onset of alcohol use, days of abstinence before study or years of alcohol use as separated dependent variables.

For these analyses, and between-groups comparisons of age by unpaired *t-*test and non-parametric data by Chi-square or Fisher tests, a two-tailed *p*-value of 0.05 was used to determine statistical significance.

SPSS Statistics version 24 (IBM Corporation, USA) and Graph Pad Prism version 7 (GraphPad Software Inc., USA) were used for statistical analyses and graphic presentations.

## Results

Socio-demographic characteristics from both groups and patterns of drug use in alcoholics are presented in Table 1.

**Table 1:** Socio-demographic characteristics and pattern of alcohol use in patients with Alcohol Use Disorder (AUD, *n* = 22) and control (non-AUD, *n* = 23).

	**Non-AUD (*****n*** = **23)**		**AUD (*n* = 22)**		***p-value***
**SOCIO-DEMOGRAPHIC CHARACTERISTICS**					
Age [mean (SD)] (min – max)		52.4 (9.9) (30 – 67)	53.9 (8.8) break (30 – 68)	t_(43)_ = −0.54	0.59
Gender n (%)	Male	23 (100%)	22 (100%)	
Years of education[mean (SD)]	Up to 5 Between 6 to 9 Between 10 to 13 Between 14 to 19	6 (26.1%) 9 (39.1%) 7 (30.4%) 1 (4.3%)	10 (45.5%) 8 (36.4%) 4 (18.2%) 0 (0.0%)	X_2_ = 2.9	0.41
Employment situationn (%)	Formal job Informal job Unemployed Retired Freelance Not reported	14 (60.9%) 5 (21.7%) 0 (0.0%) 3 (13.0%) 1 (4.3%) 0 (0.0%)	1 (4.5%) 2 (9.1%) 9 (40.9%) 3 (13.6%) 5 (22.7%) 1 (4.5%)	X_2_ = 26.2	0.0002^**^
Marital staten (%)	Single Married or common-law marriage Divorced Not reported	5 (21.7%) 16 (69.6%) 1 (4.3%) 1 (4.3%)	5 (22.7%) 11 (50.0%) 6 (27.3%) 0 (0.0%)	X_2_ = 5.5	0.14
Tobacco use n (%)	Yes No	6 (26.1%) 15 (65.2%)	12 (54.5%) 10 (45.5%)	X_2_ = 5.0	0.08
**ALCOHOL USE**					
**Amount of alcohol used (drinks/day)** [mean (SD)]		0.22 (0.52)	14.7 (10.8)	t_(21.1)_ = −6.4	<0.0001^****^
**AUDIT** [mean (SD)]		1.8 (2.6)	26.8 (7.4)	t_(25.8)_ = −15.3	<0.0001^****^
**Age at onset of alcohol use** [mean (SD)]		–	15.9 (10.8)	–	–
**Days of abstinence before study** [mean (SD)] (min–max)		–	45.1 (36.8) (15–181)	–	–
**Years of alcohol use** [mean (SD)] (min–max)		–	38.1 (8.4) (15–54)	–	–

*AUDIT, Alcohol Use Disorder Identification Test*.

** *p < 0.001*,

**** *p < 0.0001*.

AUD patients and non-AUD controls were all males and matched by age with mean of 53.9 ± 8.8 (*SD*) and 52.4 ± 9.9 (*SD*) years old, respectively. Except for employment situation (*p* = 0.0002), no other characteristics, such as schooling and marital state differed from non-AUD control group (Table 1). There were more tobacco smokers among AUD patients (54.5%) than non-AUD controls (26.1%) but with no statistically significant difference between groups (Table 1).

Non-AUD controls consumed on average 0.22 ± 0.52 alcoholic drinks per day that was significantly smaller (*p* < 0.0001) than the amount consumed by AUD patients, which was on average 14.7 ± 10.8 (*SD*) drinks per day, as expected. In addition, AUDIT scores were significantly smaller (*p* < 0.0001) in non-AUD subjects (mean of 1.8 ± 2.6 *SD*) when compared to AUD controls (mean of 26.8 ± 7.4 *SD*), as it was also expected (Table 1).

AUD patients started to use alcohol at mean age of 15.9 ± 10.8 (*SD*) years, have used it for about 38.1 ± 8.4 (*SD*) years, and were in abstinence for an average of 45.1 ± 36.8 (*SD*) (Table 1).

## ^1^H MRS

### Between-groups comparisons

The MANOVA corrected by age on the proportion of Cho (Cho/Cr) showed a significant main effect [*F*_(2, 34)_ = 4.565, *p* = 0.018], having significant difference in the between-group comparison [*F*_(1, 35)_ = 9.199, *p* = 0.005] in the LPF. AUD patients showed smaller Cho/Cr when compared to non-AUD controls (Figure 1B). No difference was found in the RPF.

For Cho/Cr in the LPF, the corrected effect size by Hedges's *g*_*s*_ for two independent samples was 1.04 (mean non-AUD = 1.02, *SD* = 0.126; mean AUD = 0.87, *SD* = 0.17).

### Multiple linear regressions (AUD patients)

#### Amount of alcohol use (drinks/day)

When considering drinks of alcohol used per day and Cho/Cr in the two different regions in AUD patients, the metabolite in the LPF accounted for 35.2% of the variance of this variable, *F*_(2, 16)_ = 5.338, *p* = 0.019, adjusted *R*^2^ = 0.35, 95% CI [−72.09, −5.81]. This metabolite showed significant zero-order correlation (*r* = −0.639, β = −0.563) with drinks/day (Figure 1C) in the LPF, showing significant (*p* = 0.003) partial effect in the full model. The Cho/Cr in the RPF was not significantly correlated with drinks/day.

#### Other patterns of alcohol use

Age at onset of alcohol use, days of abstinence before this study and years of alcohol use were not associated with Cho/Cr changes in the two PF regions in these AUD patients.

## Discussion

Ratios of Cho/Cr were found significantly reduced in the left prefrontal region of AUD patients when compared to non-AUD, as it would be expected (4).

Prefrontal region is essential to adequate executive function (19–22) and is greatly affected by alcohol and drugs of abuse (21, 22). Cho seems to be involved in the synthesis and degradation of cellular membrane (23) and its reduction supposedly occurs by a direct action of alcohol over the myelin metabolism or by detoxification process after abstinence (4).

AUD patients in our study showed reduction of Cho in prefrontal area from left hemisphere only, which could be due to a greater sensitivity of this region or perhaps to a greater difficulty of its recovery (4).

The magnitude of its clinical relevance is reflected by the effect size of 1.04, which is a large effect size according to Cohen's convention (24), meaning that about 80% of AUD patients showed mean Cho proportion in the LPF below the mean Cho proportion observed in non-AUD subjects.

The progressive reduction of Cho/Cr in the LPF was associated with increasing amount of alcohol used in drinks per day in AUD patients. Ende et al. (7) showed an opposite correlation in which higher amount of alcohol use was related to increasing choline values in frontal white matter. However, the highest amount of alcohol use in their study was of 1.9 drinks per day with no diagnosis of AUD. The amount of alcohol use in our study was much higher (from 3 up to 50, with an average of 14.7, drinks per day) and our sample was constituted by severe AUD patients with a history of 38 years of alcohol use in average. In this population, Bendszus et al. (11) also found a negative correlation between Cho/Cr in frontal lobes and the amount of alcohol consumed. Therefore, the reduced Cho/Cr in the prefrontal area, notably in the left hemisphere, may indicate excessive alcohol consumption and it may be suggestive of severe AUD.

There are differences in left and right prefrontal functionality (14, 15). They even seem to exhibit opposing lateralization (14). The left dorsolateral region, where we measured the Cho metabolite, generates a high-level task set incorporating the goals and rules when these are not provided by the external context [see (14)], process that could be compromised under reduced Cho as observed in AUD patients.

There are limitations in this study that must be mentioned. Due to heterogeneous spectral curves or inadequacy in specific metabolic curves or outlier data, some subjects were excluded, and the final sample size was reduced to 22 subjects in non-AUD and 17 in AUD groups. The use of relationships between metabolites giving non-absolute values may prevent the specific identification of the variation the metabolite, but it facilitates comparisons of results among different research centers.

In summary, rates of Cho/Cr were lower in the LPF of AUD patients when comparing to non-dependent healthy controls and this decreasing was associated with increasing daily amount of alcohol use in AUD patients, possibly indicating the harmfulness of alcohol effects in this drug use disorder.

## Author contributions

RdS, MR, and EN-P conceived of the presented idea and contributed with important theoretical and technical content. RdS coordinated the recruitment of patients, established the parameters and scheduled the MRI acquisition. TR contributed with clinical details of patients and data post-processing. TE contributed with post-processing of image data. EN-P supervised the study, ran data analysis, and organized the manuscript. EG contributed with technical knowledge and co-supervised the work. All authors discussed the results and contributed to the final manuscript.

### Conflict of interest statement

The authors declare that the research was conducted in the absence of any commercial or financial relationships that could be construed as a potential conflict of interest.
